# Consequences of Untreated Dental Caries on Schoolchildren in Mexico State’s Rural and Urban Areas

**DOI:** 10.3390/dj13080359

**Published:** 2025-08-07

**Authors:** José Cuauhtémoc Jiménez-Núñez, Álvaro Edgar González-Aragón Pineda, María Fernanda Vázquez-Ortíz, Julio César Flores-Preciado, María Eugenia Jiménez-Corona, Socorro Aída Borges-Yáñez

**Affiliations:** 1 Master and Doctoral Program in Medical-Dental and Health Sciences at the School of Dentistry, National Autonomous University of Mexico, Ciudad de México 04510, Mexico; cuauhtemoc.jimenez@iztacala.unam.mx; 2 Faculty of Higher Studies (FES) Iztacala, National Autonomous University of Mexico, Ciudad de México 54090, Mexico; mafernanda.vazquez@iztacala.unam.mx; 3 Faculty of Dentistry, Autonomous University of Baja California, Mexicali 21100, Mexico; julio.cesar.flores.preciado15@uabc.edu.mx; 4 Department of Epidemiology, Ignacio Chávez National Institute of Cardiology, Ciudad de México 14080, Mexico; mejimenez777@gmail.com; 5 Dental Public Health Department, Graduate and Research Division at the School of Dentistry, National Autonomous University of Mexico, Ciudad de México 04510, Mexico; aborges@unam.mx

**Keywords:** dental caries susceptibility, epidemiological monitoring, rural health, urban health

## Abstract

**Background/Objectives**: Dental caries is the most prevalent oral condition worldwide. Consequences of untreated dental caries (CUDC) can range from pulp damage and soft tissue ulceration due to root debris to more severe issues, such as fistulas and abscesses. Rural communities might be more vulnerable to CUDC because of lower socioeconomic status, poorer access to healthcare, and lower education levels. The objective of this study was to evaluate and compare the prevalence of CUDC in rural and urban areas in schoolchildren aged 8 to 12 years in the State of Mexico. **Methods**: A cross-sectional study was conducted using the PUFA index, considering the presence of pulp involvement (P), soft tissue ulcerations due to root remnants (U), fistulas (F), and abscesses (A). The independent variable was the geographic area (rural or urban), and the covariates were nutritional status, hyposalivation, having one’s own toothbrush, and having received topical fluoride in the last year. Logistic regression models were fitted, calculating odds ratios (ORs) and 95% confidence intervals (CIs). **Results**: The prevalence of CUDC (PUFA > 0) was 42.9% in rural areas and 25.9% in urban areas. Residing in a rural area (OR: 2.15, 95% CI 1.38–3.34, *p* = 0.001), hyposalivation (OR: 1.93, 95% CI 1.11–3.37, *p* = 0.020), and professional fluoride application (OR: 0.15, 95% CI 0.07–0.32, *p* < 0.001) were associated with the prevalence of CUDC. **Conclusions**: To prevent caries and its clinical consequences due to the lack of treatment, it is important to promote timely care seeking and access to dental care services, considering the conditions of each geographic area.

## 1. Introduction

Dental caries, also known as tooth decay, is a complex, multifactorial, non-communicable, biofilm-mediated disease, influenced by diet, that results in net mineral loss from dental hard tissues [1]. Dental caries is the most prevalent oral condition worldwide [2]. According to data from the World Health Organization (WHO), the disease affects about 45% of the population, and this number is expected to grow [3,4]. The impact of dietary sugars, biofilm, and their duration on tooth surfaces is widely recognized as a major factor in the development of cavities. However, new research highlights the link between this disease and social, economic, and behavioral factors, particularly oral hygiene [5,6,7]. This can be particularly important during the mixed dentition period, as the mouth, perhaps more than the rest of the body, is in a state of almost continuous change. This transitional phase makes maintaining dental health particularly difficult [8].

As carious lesions progress, the need for treatment increases because this condition often reduces patients’ quality of life [9] due to the painful and expensive consequences of untreated dental caries (CUDC) [10]. Knowing the clinical consequences of this condition is important to establish the impact of difficulties in accessing health services and socioeconomic status, especially in developing countries, since it has been reported that dental care in these countries is often late and scarce [11]. Indices such as the International Caries Detection and Assessment System (ICDAS) and the number of decayed, missing, and filled teeth (DMFT) provide information on caries and restorative treatment but do not provide information on CUDCs, such as pulp involvement and dental abscess, which may be more serious than caries lesions themselves [12,13]. Monse et al. [14] introduced the PUFA index to measure the occurrence and severity of oral problems due to untreated tooth decay. The index comprises pulp involvement, soft tissue ulcerations caused by root debris, fistulas, and abscesses (hence the name, PUFA).

A person’s residence (rural or urban) influences their exposure to various disease risk factors, reflecting the impact of location on lifestyle and behavior [15]. The connection between cavities and socioeconomic variables, including location, has been examined in earlier studies [16,17,18,19]. Studies indicate higher caries rates in rural areas, primarily attributed to socioeconomic factors like poverty, limited healthcare, and lower education [20]. Data suggests a correlation between urban living and better understanding of dental hygiene practices like fluoride use and regular brushing [21]. Similarly, in urban areas, there is greater access to health services compared to rural areas [22,23]. Research indicates a correlation between rural residence and higher CUDC prevalence when unfavorable sociodemographic factors are considered [24,25]. On the other hand, others have not found an association between the geographical area and the prevalence of these conditions [26,27].

The objective of this study was to evaluate and compare the prevalence of CUDC in rural and urban areas among schoolchildren aged 8 to 12 in the State of Mexico.

## 2. Materials and Methods

A cross-sectional study was conducted in public elementary schools in Acambay de Ruiz Castañeda and Tlalnepantla de Baz in the State of Mexico. Data was gathered from August 2024 to January 2025, following the parameters established by the Strengthening the Reporting of Observational Studies in Epidemiology (STROBE) Declaration.

### 2.1. Participants

The National Institute of Statistics and Geography (INEGI) classifies localities as rural or urban according to the number of inhabitants. Acambay de Ruiz Casteñeda contains the rural locality of San Antonio Detiña [28]. Conversely, within Tlalnepantla de Baz lies San Bartolo Tenayuca, which is considered an urban community. In Acambay de Ruíz (rural area), 17.1% of the population lives in extreme poverty, and 1.8% is classified as vulnerable due to their income. In contrast, Tlalnepantla de Baz’s urban area shows 15.4% in extreme poverty and 1.5% categorized as vulnerable [29]. Concerning education, Acambay shows 8% illiteracy (15+) and 8 years of average schooling, while Tlalnepantla has 1.8% illiteracy and 11 years of average schooling [28].

Sample size was determined using a Polish schoolchildren study [25] as a basis, aiming for a 5% margin of error and 95% confidence in our proportion estimate. A total of 424 schoolchildren were invited, assuming a 15% non-response rate. This study included children aged 8–12 years attending public schools in the aforementioned localities. Children with health conditions and orthodontic appliances that limited the oral assessment were excluded. Due to accessibility difficulties in rural areas, the selection of participants was by convenience.

Informed written consent was obtained from the child’s parents or guardians, and the student also assented. The protocol was evaluated and approved by the Ethics Committee of the Iztacala Faculty of Higher Studies (CE/FESI/082024/1792).

### 2.2. Variables

The prevalence of CUDCs was the dependent variable using the PUFA index criteria, considering the presence of pulp involvement (P), soft tissue ulcerations due to root debris (U), fistulas (F), and abscesses (A). The prevalence of CUDCs was determined if the participant had any of the conditions assessed by the PUFA index in at least one tooth (PUFA > 0) [14], regardless of the type of dentition (temporary or permanent). The independent variable was the geographic area (rural/urban), and the covariates were nutritional status (underweight/normal weight/overweight/obesity), hyposalivation (no/yes), having their own toothbrush (no/yes), and having received topical fluoride in the last year (no/yes). Information was also collected on age (completed years), sex (male/female), diet (consumption of artificial juices, soft drinks, sweets and chewing gum at least once a week [no/yes]), consumption of sugary drinks before bedtime (no/yes), oral hygiene (Simplified Oral Hygiene Index [OHI-S] score) [30], tooth brushing frequency (once/twice or more), toothpaste use (no/yes), and caries experience (DMFT) [12].

### 2.3. Data Collection

#### 2.3.1. Questionnaires

To gather data on diet and hygiene, a prior study’s questionnaire was used in directed interviews [31]. The completion of this questionnaire was carried out in the classroom with the help of trained personnel to direct the correct approach and not influence the participants’ responses.

#### 2.3.2. Oral Health Assessment

Dental assessment was performed by an expert-standardized examiner using PUFA, OHI-S, and DMFT indices (kappa > 0.80). Existing teeth were assessed, regardless of whether they were primary or permanent teeth. Oral examinations were conducted in a classroom at the schools visited. For dental assessment, participants lay on a folding table. Artificial light was used to illuminate the mouth, along with a dental mirror (Araín, Sialkot, Punjab, Pakistan) and a PCP11 probe (Hu-Friedy, Chicago, IL, USA). The probe was only used to assess oral hygiene; for the PUFA index, only the dental mirror was used. First, oral hygiene was assessed using the OHI-S [30]; then, soft matter was removed from all dental organs, and the presence of CUDC was evaluated by identifying pulp involvement (P), ulcerations (U), fistulas (F), and abscesses (A): presence = existence of the condition, and absence = no such conditions exist [14]. Likewise, information was collected from the DMFT index in terms of the number of decayed, missing, and filled teeth [12]. Hyposalivation was assessed by the wafer test, for which a wafer was placed on the back of the tongue, and the time it took for the wafer to dissolve was calculated (> 4 min = hyposalivation) [32].

#### 2.3.3. Nutritional Assessment

For nutritional status, with the help of a Seca^®^ model 213 portable stadiometer and a Tanita^®^ model BC-558 segmental composition monitor, the weight and height of the schoolchildren were measured. The Body Mass Index (BMI) was calculated using the AnthroPlus software(Version 3.2.2) to categorize the nutritional status BMI for age z scores (BAZ): BAZ < −2 = underweight, BAZ −1.99–0.99 = normal weight, BAZ 1–1.99 = overweight, and BAZ > 2 = obesity [33].

### 2.4. Data Analysis

Data was analyzed using Stata V.17 software (Stata Corp., College Station, TX, USA). Frequencies and percentages were obtained for qualitative variables (sex, CUDC, geographic area, nutritional status, hyposalivation, having one’s own toothbrush, topical fluoride, diet, brushing frequency, and toothpaste use), and central tendency measures were obtained for quantitative variables (age, oral hygiene, and caries experience). Bivariate analysis was performed according to geographic area and CUDC prevalence, using the Chi square (X^2^) or Fisher’s exact test for categorical data and the Mann–Whitney U test for quantitative data. In the saturated model, a *p*-value < 0.05 was considered statistically significant. Logistic regression models were fitted for multivariate analysis, having as dependent variable the CUDC. Variables with a *p*-value < 0.25 were included, as well as those relevant based on biological plausibility. Odds ratios (ORs) and 95% confidence intervals (CIs) were calculated. Confounding and interactions were also assessed.

## 3. Results

### 3.1. Population Characteristics by Geographical Area

This study comprised 408 schoolchildren (203 rural and 205 urban) with a 3.7% non-response rate (16 out of 424). On average, participants were 10.01 ± 0.93 years old, with females comprising 52.7% of the sample. The schoolchildren had an average of 22.34 ± 2.63 teeth, with 4.60 ± 4.07 primary teeth and 17.74 ± 5.16 permanent teeth.

In urban areas, there was a higher consumption of soft drinks (38.1% vs. 26.2%, *p* = 0.010), while in rural areas, there was a higher consumption of sugary drinks before bedtime (11.8% vs. 7.4%, *p* = 0.012). Schoolchildren in urban areas reported higher brushing frequency (87.8% vs. 72.0%), toothpaste use (100% vs. 97.6%), and showed lower OHI-S (1.1 ± 0.5 vs. 1.7 ± 0.6) (*p* < 0.05).

Rural areas showed significantly more decayed teeth (5.9 ± 3.0) than urban areas (4.6 ± 3.0) according to the DMFT index (*p* < 0.001). This study revealed a statistically significant difference (*p* < 0.001) in caries prevalence between rural (92.6%) and urban (82.5%) areas, with an overall prevalence of 87.5%. A statistically significant difference (*p* = 0.041) was found in the prevalence of filled teeth, with a higher percentage in urban areas (37.1%) than in rural areas (27.6%). Table 1 details the age, sex, diet, dental hygiene, and caries experience of the school children who were part of this study.

### 3.2. Consequences of Untreated Dental Caries

**Figure 1:**
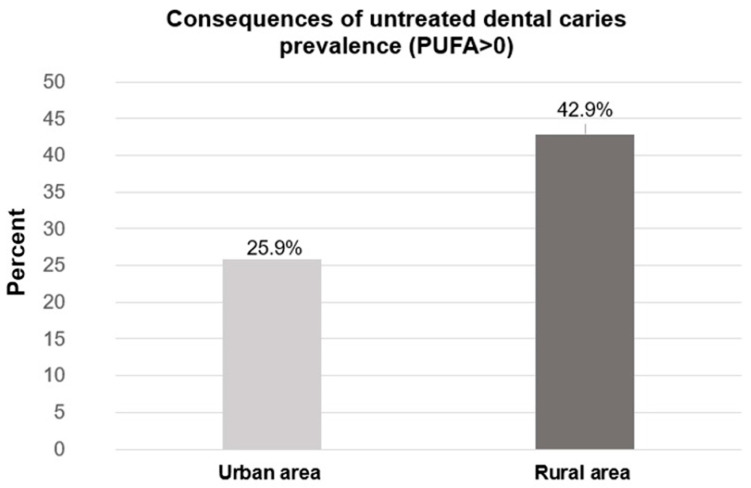
Prevalence of consequences of untreated dental caries (CUDC) according to the geographical area in the group of schoolchildren from the State of Mexico who participated in this study.

Regarding CUDC, 34.3% of the sample presented at least one of these conditions (PUFA > 0), being more frequent in rural areas (42.9%) than in urban areas (25.9%) (*p* = 0.007) (Figure 1). Within the specific clinical consequences related to untreated caries, a higher prevalence of fistulas (30.1% vs. 14.2%, *p* < 0.001) and abscesses (30.5% vs. 16.5%, *p* = 0.001) was found in rural areas than in urban areas. Rural and urban CUDC are displayed in Table 2.

Schoolchildren who reported having received fluoride in the past year had a lower prevalence of CUDC (10.8% vs. 40.4%, *p* < 0.001) (Table 3).

### 3.3. Multivariate Analysis

The logistic regression model included geographic area, nutritional status, hyposalivation, own toothbrush, and topical fluoride application, adjusted for age and sex. The final model showed that residing in a rural area had an OR = 2.15 for developing CUDC (95% CI 1.38–3.34, *p* = 0.001). Having hyposalivation OR = 1.93 (95% CI 1.11–3.37, *p* = 0.020) and reporting professional application of topical fluoride reduced the odds ratio for developing CUDC by 85% (OR: 0.15, 95% CI 0.07–0.32, *p* < 0.001). The final model with the crude and adjusted odds ratios is presented in Table 4.

## 4. Discussion

This study found a greater prevalence of caries in schoolchildren living in rural areas (5.9 teeth) than in those living in urban areas (4.6 teeth). Similarly, a higher prevalence of CUDC was found in schoolchildren living in rural areas (42.9%) than in those living in urban areas (25.9%). These results highlight the need to develop intervention strategies tailored to the care needs of each population based on their area of residence. Living in a rural area is a risk indicator for experiencing some of the clinical consequences resulting from a lack of dental care.

To our knowledge, this study is the first to compare the prevalence of CUDC caries in Mexico. Additionally, it is the first to contrast the frequency of clinical issues from untreated cavities in the Americas. Among the weaknesses of this study is that, given its cross-sectional nature, causality cannot be established. Conversely, convenience sampling means generalizations should be treated with caution. Finally, since this was an epidemiological study, the use of radiographic images as a diagnostic aid was beyond our scope.

The overall prevalence of CUDC reported in different studies ranges from 7.2% to 84% in schoolchildren [14,24,34,35]. In this study, a prevalence of 34.3% was found. According to the geographic area, this study found an association between residing in a rural area and a higher prevalence of CUDC, results that are similar to those found in two studies conducted in schoolchildren in India according to the PUFA index [24,25]. On the other hand, two studies conducted in Nepal and the Philippines, which used this same index when comparing these two population groups, did not find an association. However, they highlight the importance of studying this condition from a sociodemographic approach due to the evident centralization of dental services in urban areas of these countries [26,27]. The dental profession must provide tools for healthcare decision-making based on disease levels. The index defines four different clinical stages of advanced caries, offering greater information based on the CUDC. Using the PUFA index to present data gives healthcare planners useful information that adds to what the ICDAS and DMFT indices provide [14].

The probability of suffering from any of the CUDCs in schoolchildren in the State of Mexico increased in those who resided in rural areas (OR: 2.15). This could be explained by the social and economic determinants that condition a low income and a high poverty rate in these areas. Limited access to oral healthcare results in more frequent and severe oral diseases due to insufficient prevention and timely treatment [36]. This also has to do with the proposed policies, in which oral care centers in rural areas are scarce and provide a limited number of services that ignore the care needs of the population [37]. In these areas, the limited availability of education—a key source of social and academic resources—significantly affects people’s health and their adoption of self-care practices [38].

Schoolchildren with hyposalivation showed a higher probability of presenting CUDC (OR: 1.93). Saliva is essential to remineralize dental tissues by providing calcium, phosphate, and fluoride [39], so in subjects with carious lesions, the reduction of saliva production can benefit cariogenic microorganisms to continue aggravating them and present CUDC [40]. The lack of saliva can benefit the accumulation of bacterial colonies, generating a dysbiotic environment favorable for the progression of demineralization of the tooth surface and progression of cavities [41,42].

Finally, it was found that professional application of fluoride (OR: 0.15) was a protective factor against the prevalence of CUDC. The fluoride levels that can be achieved with the use of topical gels or varnishes are higher than other sources [43]. Fluoride is a compound that decreases the solubility of tooth enamel by incorporating itself into hydroxyapatite crystals, thereby decreasing their solubility and pH, which is critical for their dissolution. Fluoride also exerts its anticariogenic action by being in solution and modifying the saturation characteristics of the dental mineral in the biofilm fluid, both on the dental surface and within the dental mineral, by promoting remineralization and decreasing demineralization, and, in sufficient concentration, by inhibiting bacterial carbohydrate metabolism [44,45].

In the development of this study, it became evident that caries-related conditions and the clinical consequences of neglecting them depend not only on primary etiological factors but also on the social structural determinants of health, such as geographic area, which limit the prevention, diagnosis, and treatment of dental caries. To improve oral health, future planning must consider area-specific (rural/urban) interventions.

## 5. Conclusions

The prevalence of CUDC was higher in rural areas than in urban areas. To prevent caries and its clinical consequences due to the lack of treatment, it is important to promote timely care seeking and access to dental care services, considering the conditions of each geographic area. The results of this study highlight the importance of addressing the needs of minors as a vulnerable population, in addition to the disadvantages of rural areas.

## Figures and Tables

**Table 1:** Description of the school groups considered in this study (age, sex, diet, dental hygiene, and caries experience) according to the geographical area.

		Total (%) *n* = 408 (100.0)	Rural (%) *n* = 203 (100.0)	Urban (%) *n* = 205 (100.0)	*p*
Age	Mean ± S.D.	10.0 ± 0.9	9.9 ± 0.8	10.0 ± 0.9	0.052 *
Sex	Male	193 (47.3)	103 (50.7)	90 (43.9)	0.167 **
	Female	215 (52.7)	100 (49.3)	115 (56.1)	
Artificial juice intake	No	300 (73.5)	155 (76.3)	145 (70.7)	0.198 **
	Yes	108 (26.5)	48 (23.7)	60 (29.3)	
Soft drink consumption	No	277 (67.8)	150 (73.8)	127 (61.9)	0.010 **
	Yes	131 (32.2)	53 (26.2)	78 (38.1)	
Consuming sugary drinks before bed	No	369 (90.5)	179 (88.2)	190 (92.6)	0.012 **
	Yes	39 (9.5)	24 (11.8)	15 (7.4)	
Intake of candy	No	268 (65.6)	130 (64.1)	138 (67.4)	0.468 **
	Yes	140 (34.4)	73 (35.9)	67 (32.6)	
Chewing gum consumption	No	327 (80.2)	158 (77.8)	169 (82.4)	0.243 **
	Yes	81 (19.8)	45 (22.2)	36 (17.6)	
Brushing frequency	Once a day	82 (20.1)	57 (28.0)	25 (12.2)	0.001 **
	At least twice a day	326 (79.9)	146 (72.0)	180 (87.8)	
Use of toothpaste	No	5 (1.3)	5 (2.4)	0 (0.0)	0.024 ***
	Yes	403 (98.7)	198 (97.6)	205 (100.0)	
OHI-S	Mean ± S.D.	1.4 ± 0.6	1.7 ± 0.6	1.1 ± 0.5	0.001 *
DMFT	Mean ± S.D.	5.2 ± 3.1	5.9 ± 3.0	4.6 ± 3.0	<0.001 *

S.D. = standard deviation; OHI-S = Simplified Oral Hygiene Index; DMFT = Decayed, Missing, and Restored Teeth Index; * Mann–Whitney U statistical test; ** Chi square statistical test; *** Fisher’s exact statistical test.

**Table 2:** Consequences of untreated dental caries (CUDC) according to the geographical area in the group of schoolchildren from the State of Mexico who participated in this study.

		Total (%) *n* = 408 (100.0)	Rural (%) *n* = 203 (100.0)	Urban (%) *n* = 205 (100.0)	*p*
Pulp involvement (P)	No	272 (66.6)	126 (62.0)	146 (71.2)	0.050 *
	Yes	136 (33.4)	77 (38.0)	59 (28.8)	
Ulceration (U)	No	405 (99.2)	200 (98.5)	205 (100.0)	0.071 **
	Yes	3 (0.8)	3 (1.5)	0 (0.0)	
Fistula (F)	No	318 (77.9)	142 (69.9)	176 (85.8)	<0.001 *
	Yes	90 (22.1)	61 (30.1)	29 (14.2)	
Abscess (A)	No	312 (76.4)	141 (69.5)	171 (83.4)	0.001 *
	Yes	96 (23.6)	62 (30.5)	34 (16.5)	
CUDC (PUFA)	No	268 (65.7)	116 (57.1)	152 (74.1)	0.007 *
	Yes	140 (34.3)	87 (42.9)	53 (25.9)	

PUFA = pulp involvement, ulcerations, fistula, and abscess; * Chi square statistical test; ** Fisher’s exact statistical test.

**Table 3:** Nutritional status, hyposalivation, having their own toothbrush, and application of topical fluoride, according to the consequences of untreated dental caries (CUDC) in the group of schoolchildren from the State of Mexico who took part in this study.

		Total (%) *n* = 408	CUDC = 0 (%) *n* = 268	CUDC > 0 *n* = 140	*p*
Nutritional status	Underweight	8 (100.0)	5 (62.5)	3 (37.5)	0.212 *
	Normal weight	209 (100.0)	134 (64.1)	75 (35.9)	
	Overweight	97 (100.0)	59 (60.8)	38 (39.2)	
	Obesity	94 (100.0)	70 (74.4)	24 (25.6)	
Hyposalivation	No	385 (100.0)	257 (66.8)	128 (33.2)	0.063 **
	Yes	23 (100.0)	11 (47.8)	12 (52.2)	
Own toothbrush	No	34 (100.0)	21 (61.7)	13 (38.3)	0.615 **
	Yes	374 (100.0)	247 (66.0)	127 (34.0)	
Topical fluoride application	No	324 (100.0)	193 (59.6)	131 (40.4)	<0.001 **
	Yes	84 (100.0)	75 (89.2)	9 (10.8)	

* Fisher’s exact statistical test. ** Chi square statistical test.

**Table 4:** Odds ratios and confidence intervals of the logistic regression models for the consequences of untreated dental caries according to the geographic area and covariates in the group of schoolchildren from the State of Mexico who participated in this study.

Variables	Crude OR (CI 95%)	Adjusted OR * (CI 95%)	Age and Sex Adjusted OR (CI 95%)
Geographic area (reference = urban)	2.19 (1.42–3.56)	2.16 (1.39–3.42)	2.15 (1.38–3.34)
Hyposalivation (reference = no)	2.06 (1.24–4.23)	2.01 (1.19–3.51)	1.93 (1.11–3.37)
Application of topical fluoride (reference = no)	0.18 (0.12–0.52)	0.16 (0.09–0.46)	0.15 (0.07–0.32)

OR = odds ratio; CI = confidence Interval; * Includes geographic area, hyposalivation, and application of topical fluoride without adjusting for age and sex.

## Data Availability

The original contributions presented in this study are included in this article. Further inquiries can be directed to the corresponding author.

## Abbreviations

The following abbreviations are used in this manuscript:

CUDC	Consequences of untreated dental caries
PUFA	Pulp involvement, ulcerations, fistula, and abscess
OR	Odds ratio
CI	Confidence interval
DMFT	Number of decayed, missing, and filled teeth
ICDAS	International caries detection and assessment system
OHI-S	Simplified Oral Hygiene Index
BMI	Body mass index
